# *In vitro* impact of platinum nanoparticles on inner ear related cell culture models

**DOI:** 10.1371/journal.pone.0284794

**Published:** 2023-04-24

**Authors:** Elisabeth Berger, Gudrun Brandes, Janin Reifenrath, Thomas Lenarz, Martin Durisin, Kirsten Wissel

**Affiliations:** 1 Hannover Medical School, Department of Otorhinolaryngology, Hannover, Germany; 2 Hannover Medical School, Lower Saxony Centre for Biomedical Engineering, Implant Research and Development (NIFE), Hannover, Germany; 3 Hannover Medical School, Institute of Neuroanatomy and Cell Biology, Center of Anatomy and Cell Biology, Hannover, Germany; 4 Hannover Medical School, Clinic for Orthopaedic Surgery, Hannover, Germany; 5 University Clinic of Otolaryngology, Head and Neck Surgery, Otto-von-Guericke-University Magdeburg, Magdeburg, Germany; Xiangtan University, CHINA

## Abstract

So far, it was supposed that the increase of electrical impedance following cochlear implant (CI) insertion was due to technical defects of the electrode, inflammatory and/or formation of scar tissue along the electrode. However, it was recently reported that corrosion of the platinum electrode contacts may be the reason for high impedances. It could be shown that platinum particles were stripped from the electrode surfaces. Its potential cytotoxic effects within the inner ear remains to be examined. In this study *in vitro* cell culture models of the mouse organ of Corti cell line (HEI-OC1) and the spiral ganglion (SG) cells derived from the cochleae neonatal rats were used to investigate the effects of the polyvinylpyrrolidone coated platinum nanoparticles (Pt-NP_PVP_, 3 nm) on cell metabolism, neuronal survival and neurite outgrowth. Our data revealed no decrease of the metabolic activity of the HEI-OC1 cells at Pt-NP_PVP_ concentrations between 50–150 μg/ml. Also, staining with Calcein AM/EthD demonstrated prevalent presence of vital cells. As shown by transmission electron microscopy no Pt-NP_PVP_ could be found at the cell surface or in the cytosol of the HEI-OC1 cells. Similarly, the SG cells exposed to 20–100 μg/ml Pt-NP_PVP_ did not show any reduced survival rate and neurite outgrowth following staining of the neurofilament antigen even at the highest Pt-NP_PVP_ concentration. Although the SG cells were exposed to Pt-NP_PVP_ for further 72 h and 96 h immunocytochemical staining of the glial cells and fibroblasts presented normal cell morphology and growth independently of the cultivation period. Our data indicates that the used Pt-NP_PVP_ do not trigger the cellular uptake and, thus, presumable do not initiate apoptotic pathways in cells of the organ of Corti cell line or the auditory nerve. The protection mechanisms to the Pt-NP_PVP_ interactions remain to be clarified.

## Introduction

Insertion of the cochlea implant (CI) into the scala tympani evokes electrode insertion trauma resulting in an increase of impedance due to mechanical damage of the lateral wall and basilar membrane [1–5] and induction of inflammation processes [6–8]. Consequently, fibrosis on the implant surface and new bone formation inside the scala tympani [4, 5, 9–12] were observed reducing hearing benefit and in some cases leading to technical failure of the implant [13, 14]. In addition, explantation of such implants revealed eroded platinum electrode surfaces [15–17]. As the consequence of faradeic processes Pt^2+^ ions and their chloride oxidation products as like as ClO^-^ and ClO^3-^ appeared as the most likely dissolved species at the Pt/saline interface [18, 19]. Additionally, traces of particulate and nano sized Pt were also demonstrated in animals following electrical stimulation for several weeks [20–22] and long-term CI users [16, 23–25]. They have been mainly detected in the electrode-tissue capsules mostly phagocytosed by macrophages, however, they were also found in the human spiral ligament [24]. Release of Pt particles have been found in all cochlear specimens examined so far implying that corrosion of the Pt electrode contacts is relatively widespread in cochleae of long-term CI patients–independently of a specific implant design [16, 23–25]. Furthermore, it could be shown that the tissue response including development of focal necrosis at the electrode-tissue-interface and the extent of corrosion are related to increasing charge densities and not to the amount of Pt particles released from the electrode contacts [26]. It is speculated that ionic Pt generated as Pt + 6Cl^−^ ⇔ [PtCl_6_]^-2^ + 4e− under anodic conditions may be reduced to elementary Pt and redeposited during the cathodic pulse [18, 27–29]. Whereas any measurable adverse effects on cochlear tissue in neither explanted CI-patients [16, 23–25] nor animal models [20–22] were observed, controversial effects of Pt nanoparticles (Pt-NP) were found in *in vitro* cell culture models on defined cell types. Some reports confirmed any cytotoxic effects of Pt-NPs with average size below 10 nm and at concentrations below 100 μg/ml in several human and animal cell lines [30–36]. Furthermore, Pt-NP are very well characterized as reactive oxygen species scavenger [30, 37–41]. On the other hand, it has also been reported that 1–9 nm sized Pt-NP induced oxidative stress, inflammation, chromosome condensation or other negative biological effects in a concentration depending manner in normal and cancer cells of both human and animals, bacteria and algae [31, 35, 42–49]. In a previous study using Pt-NP_PVP_ industrially manufactured with 3 nm in size, the viability of the NIH 3T3 and human neuroblastoma cells (SH-SY5Y) was clearly reduced depending on the concentration of the Pt-NP_PVP_ [50].

The aim of this study was to investigate the potential cytotoxicity of Pt-NP_PVP_ in specific cells of the inner ear (Table 1). For the first time, primary cells from the neonatal rat spiral ganglions (SG cells) and the immortalized mouse organ of Corti cell line (HEI-OC1) were exposed to different concentrations of Pt-NP_PVP_. The authors hypothesized that these inner ear-specific cells would show a comparable decrease in viability after addition of Pt-NP_PVP_ as detected in non-specific inner ear cells NIH 3T3 and SH-SY5Y exposed to the same Pt-NP_PVP_ [50]. The effects of Pt-NP_PVP_ in the HEI-OC1 and SG cells were characterized by determining the mitochondrial activities, extent of apoptotic cells, survival rates and neurite outgrowth, SG cell population composition as well as cell morphology and ultrastructure of the cells.

**Table 1:** Cell culture models of the inner ear that have been used in this study.

Description	Appreciation	Origin
Immortalized mouse organ of Corti cell line	HEI-OC1	House Ear Institute-Organ of Corti 1
Spiral ganglion cells containing glia cells, neurons and fibroblasts	SG cells	Neonatal rats
Spiral ganglion neurons alone	SG neurons	Neonatal rats

## Methods

### Dispersion of the platinum nanoparticles coated with polyvinylpyrrolidone (Pt-NP_PVP_)

A 40 mg/ml stock solution of hydrophilic Pt-NP_PVP_ powder (particle size: 3 nm, PlasmaChem, Berlin, Germany) was dispersed in sterile Aqua bidest. by water bath sonication for 15 min. The Pt-NP_PVP_ stock solution was diluted to the desired concentrations in high glucose Dulbecco’s Modified Eagle’s Medium (DMEM, Bio&Sell, Germany) completed with 10% fetal calf serum (FCS, Bio&Sell, Germany). According to Kalinec et al. the cell culture medium was not supplemented with any antibiotics [51].

### Seeding and cultivation of the organ of Corti cell line (HEI-OC1) following Pt-NP_PVP_ supplementation

The HEI-OC1 cell line was kindly provided by Michael Morgan (Institute of Experimental Hematology, Hannover Medical School, Hannover, Germany). Prior to Pt-NP_PVP_ application 4000 cells were seeded in 96-well plates each containing 100 μl of the cell culture medium (high glucose DMEM and 10% FCS) and pre-cultured under permissive conditions (33°C, 10% CO_2_) for 24 h. As follows the cell culture medium was exchanged with those containing Pt-NP_PVP_ concentrations between 50 μg/ml and 150 μg/ml and the cells were cultivated for further 48 h. For statistical assessment at least N = 6 experiments were considered and each cell culture assay was prepared in triplicates. To enable relative quantification of samples cultivated in different Pt-NP_PVP_ concentrations HEI-OC1 cells without any treatment were used as reference, additionally cells exposed to 15% DMSO for 1.5 h were included to the study as negative control. Light microscopy (Olympus CKX41SF, Hamburg, Germany) was used to monitor changings in density and the morphology of the HEI-OC1 cells and images were taken digitally by the CCD colour camera (Olympus Color View III, Hamburg, Germany).

### Relative quantitative determination of the effects of Pt-NP_PVP_ on the metabolic activity of the HEI-OC1 cells using the fluorescently active resazurin assay

Relative quantification of changings in mitochondrial activity potentially induced by Pt-NP_PVP_ was performed by using the VisionBlueTM Quick Cell Viability Fluorometric Assay Kit (BioVision, Mountain View, CA, USA). The compound resazurin is a water-soluble, blue, non-fluorescent redox dye that is irreversibly converted to the pink, highly fluorescent resorufin upon reduction conducted by dehydrogenases in normally functioning cells. Cytotoxic substances lower the metabolic activity and redox potential of cells, leading to slowing down or termination of the resazurin reduction. Depending on the toxic potency of the substance the signal intensities—measured as fluorescence units—are proportional to the metabolic activity of the samples. The 10% resazurin solution was prepared with fresh medium and applied to the samples as described by the manufacturer followed by the incubation at 33°C for 2.5 h. Absorbance was measured at 550/600 nm (excitation/emission wave length) using a microplate reader (Synergy H1, Biotek, Bad Friedrichshall, Germany). Resazurin solution prepared with fresh medium was used as a background control. For data assessment the signal intensities received from the Pt-NP_PVP_ treated samples were related to those of the reference and calculated in percent (%). According to ISO 10993–5:2009 cell viability or metabolic activity of less than 70% in relation to the reference was considered cytotoxic [52].

### Ethidium homodimer III (EthD) and calcein acetoxymethyl (Calcein AM) labelling of the HEI-OC1 cells exposed to Pt-NP_PVP_ for qualitative examination of the cell viability

To distinguish between live and dead cells in the same cell population following Pt-NP_PVP_ administration the viability/cytotoxicity assay kit (Biotium, Fremont, CA, USA) containing Calcein AM (green) and EthD (red) was used. Calcein AM is a membrane-permeant, non-fluorescent substrate cleaved by the esterases in the cytoplasm of living cells. The cleavage product calcein turns to the green fluorescent dye by complexing Ca^2+^-ions. Since it is not able to permeate the cell membrane, it retained in the cytoplasm of viable cells with intact plasma membranes. In contrast, EthD is a DNA intercalating dye that only enter dead cells, when their plasma membrane becomes leaky.

For staining viable and apoptotic HEI-OC1 cells in the same population the assay was performed accordingly to the manufacturer’s protocol with slight modifications. Briefly, 2 μM Calcein AM and 4 μM EthDIII were prepared in serum-free high glucose DMEM. 30 μl of the staining solution was added to 100 μl of the cell culture assays without medium exchange, followed by incubation for 30–45 minutes at room temperature and fluorescent microscopic evaluation of the HEI-OC1 cells (Keyence BZ 9000 Biorevo, Keyence International, Mechelen, Belgium).

### Ethics statement

The preparation of SG cells and SG neurons used for the experiments and analyses in this study were performed in accordance with the institutional guidelines for animal welfare of the Hannover Medical School, corresponding to the standards as defined by the German ‘Animal Welfare Act’ (Tierschutzgesetz) and with the European Directive 2010/63/EU for protection of animals used for experimental purposes. The use of animals exclusively for tissue analyses is registered (no.: 2018/215) by the local authorities (Zentrales Tierlaboratorium, Laboratory Animal Science, Hannover Medical School, including an institutional animal care and use committee) and regularly reported as required by law. No further authorisation is required if no other treatment is performed prior to the killing (§4, Animal Welfare Act).

### Dissection and dissociation of spiral ganglions containing neurons, fibroblasts and glial cells

Neonatal Sprague-Dawley rats (P3-5, n = 10–12 animals) were used for dissection of the spiral ganglions. The rats were decapitated with sharp scissors and the skin of the heads was bluntly cut off from the skull. After removal of the mandibula and the skin, the skull was opened along the midline and separated into two halves. The brain was removed and the two head halves were immersed in ice-cold PBS (Invitrogen, Karlsruhe, Germany). Further dissection was performed under microscopic view (Leica MZ-6, Bensheim, Germany). The bony cochlear capsule was carefully opened and the stria vascularis and organ of Corti were removed from the modiolus. Subsequently, the entire spiral ganglions were dissected from the modiolus and transferred to ice-cold Ca^2+^/ Mg^2+^-free Hank’s balanced salt solution (HBSS, Invitrogen, Darmstadt, Germany).

The spiral ganglions were enzymatically dissociated as described by Berkingali et al. [53] with few modifications: Briefly, they were digested in 2 ml pre-warmed digestion solution containing Ca^2+^/Mg^2+^-free HBSS (Invitrogen, Karlsruhe, Germany), 0.1% trypsin (Serva, Heidelberg, Germany) and 0.01% DNase I (Roche, Mannheim, Germany) for 10–12 min at 37°C. The enzymatic activity was stopped by adding 200 μl FCS (Bio&Sell, Feucht, Germany). Following removal of the supernatant, cell clusters were washed three times in 1 ml serum-free neuromedium [Panserin 401 (PAN Biotech GmbH, Aidenbach, Germany), 1 M HEPES (Invitrogen, Karlsruhe, Germany), 10 mg/ml PBS (Invitrogen), 30 iE/ml penicillin (Sigma-Aldrich, Taufkirchen, Germany), 30% glucose in PBS (Invitrogen), 4 mg/ml insulin (Sigma-Aldrich), 1x N2 supplement (Invitrogen)]. The cell clusters were mechanically disrupted by pipetting up and down the suspension using 1000 μl and 200 μl filter tips (StarLab, Ahrensburg, Germany), respectively. Prior to the cell count using the Neubauer cell counting chamber, the cells were stained with 10% trypan blue (Sigma Aldrich) to exclude apoptotic cells from cell counting.

### Seeding and cultivation of spiral ganglion (SG) cells exposed to varying Pt-NP_PVP_ concentrations

Prior to spiral ganglion cell seed the 96-well microtiter plates were coated with each 50 μl/well of Poly-DL-Ornithine (0.1 mg/ml) for 1 h at room temperature and 0.01 mg/ml laminin (Invitrogen) for 1 h at 37°C as described previously [54]. Following cell seed (10.000 cells in 50 μl serum-free neuromedium) Pt-NP_PVP_ were applied to the samples in concentrations between 20 μg/ml and 100 μg/ml in neuromedium supplemented with 10% FCS in its final concentration. Untreated cells growing in FCS containing neuromedium served as reference for relative quantification of the survival and neurite lengths of the SG cells. Those supplemented with 2.5% DMSO were included to this study as negative control. The cultivation assays were prepared each in triplicates and at least N = 6 independent experiments were performed at 37°C and 5% CO_2_ for 48 h followed by fixation with 1:1 methanol/acetone. In addition, to determine potential time-dependent cytotoxicity of the Pt-NP_PVP_ the SG cells were exposed to varying Pt-NP_PVP_ concentrations for 72 h and 96 h also.

### Immunocytochemical determination of SG neuron survival rate, neurite outgrowth and the spiral ganglion cell composition

To determine the survival rate and neurite outgrowth of the SG neurons and the composition of the population of the spiral ganglions following concentration and time dependent Pt-NP_PVP_ exposure, immunocytochemical staining of cell specific antigens was performed. Tables 2 and 3 represent the primary and secondary antibodies used in this study. Briefly, the fixated cells were incubated with cell specific monoclonal and polyclonal antibodies diluted in 1% bovine serum albumin (BSA, Serva, Heidelberg, Germany) in PBS for 1 h at room temperature as assigned in Table 2. After washing with PBS three times for 3 min each the specific antigen-antibody interactions were detected by incubation with DAPI (Prolong^®^ anti-fade Gold with DAPI, Invitrogen) and fluorescently labelled secondary antibodies (Table 3) diluted 1:1000 and 1:400, respectively, in 1% BSA/PBS for 1 h at room temperature in darkness. The specificity of the immune staining was previously verified by omission of the primary antibodies within the light exposure range between 1.5 and 2 sec [55]. The washing steps were performed as described above. Positively stained antigens were visualised by fluorescence microscopy (Keyence BZ 9000 Biorevo, Keyence International, Mechelen, Belgium). Neurofilament staining was detected by using the fluorescence microscope (Zeiss Axio Observer Z1, Zeiss, Jena, Germany), images were digitally captured (Axiocam MRm, Zeiss, Jena, Germany) and analysed by using Palm Robosoftware (Palm Zeiss, Munich, Germany). Survival rates refer to the number of SG neurons exposed to Pt-NP_PVP_ in comparison to those of the reference (in %). For that SG neurons with neurites of at least three times the average cell diameter were included for data collection. Neurite outgrowth was determined by measuring the 5 longest neurites selected for statistical analysis.

**Table 2:** Primary antibodies for immunostaining of cell specific antigens in the SG cells.

Primary antibody	Host	Description	Specificity	Manufacturer	Dilution
Neurofilament 200 kD, monoclonal	Mouse	Intermediary filament	Neurons	Novocastra #NCL-NF200	1: 400
P75, polyclonal	Rabbit	Neurotrophic growth	Glial cells	Abcam #38335	1: 500
Vimentin clone V9, monoclonal	Mouse	Intermediary filament	Fibroblasts Glia cells	Dako #M0725	1: 200

**Table 3:** Secondary antibodies used in this study.

Secondary antibody IgG (H+L)	Host	Description	abs/em [nm]	Manufacturer
Anti-mouse	Goat	New Dylight 488 (GaM 488)	493/518	Jackson-Immunoresearch #115-485-008
Anti-rabbit	Goat	Alexa Fluor 594	591/616	Jackson-Immunoresearch #111-515-144

### Transmission electron microscopy

For investigation of the effects of Pt-NP_PVP_ on the cellular ultrastructures the HEI-OC1 cells were seeded into a 6-well microtiter plate (Nunclon, Thermo Fisher Scientific, Kempen, Germany) at densities of 300.000 cells per well and cultivated in 3 ml supplemented high glucose DMEM for 24 h as described above. As follows, the cells were cultivated in 3 ml culture medium containing 50–150 μg/ml Pt-NP_PVP_. Untreated cells in the medium alone served as controls. After 48 hours of incubation, the medium was removed and replaced with PBS. The cells were collected with the help of a cell scraper and centrifuged at 1000 rpm for 4 min (MiniSpin Plus, Eppendorf, Hamburg, Germany). The supernatant was discarded and the cell pellet was fixed with 2.5% glutardialdehyde (Polysciences, Warrington, PA, USA) in 0.1 M sodium cacodylate (Th. Geyer, Hamburg, Germany). After postfixation with 2% osmium tetroxide (Polysciences) in 0.1 M sodium cacodylate the cell pellets were embedded in epoxide resin (Serva, Heidelberg, Germany). Ultra-thin sections stained with 2% uranyl acetate (Serva) and lead citrate (Serva) were examined with the transmission electron microscope (Morgagni 268, 80 kV, Eindhoven, Netherlands). The digital images were processed with Adobe Photoshop CS6.

### Statistical analysis

All data achieved from the *in vitro* cell culture assays were presented as mean ± standard error of mean (SEM). One way nonparametric analysis of variance (ANOVA) and Tukey`s multiple comparison tests were used for statistical assessment. P < 0.05 was used as the threshold for significance in all statistical analyses.

## Results

### Exposure to Pt-NP_PVP_ did not have any impact on the morphology, ultrastructures and metabolic activity of the HEI-OC1 cells

**Fig 1:**
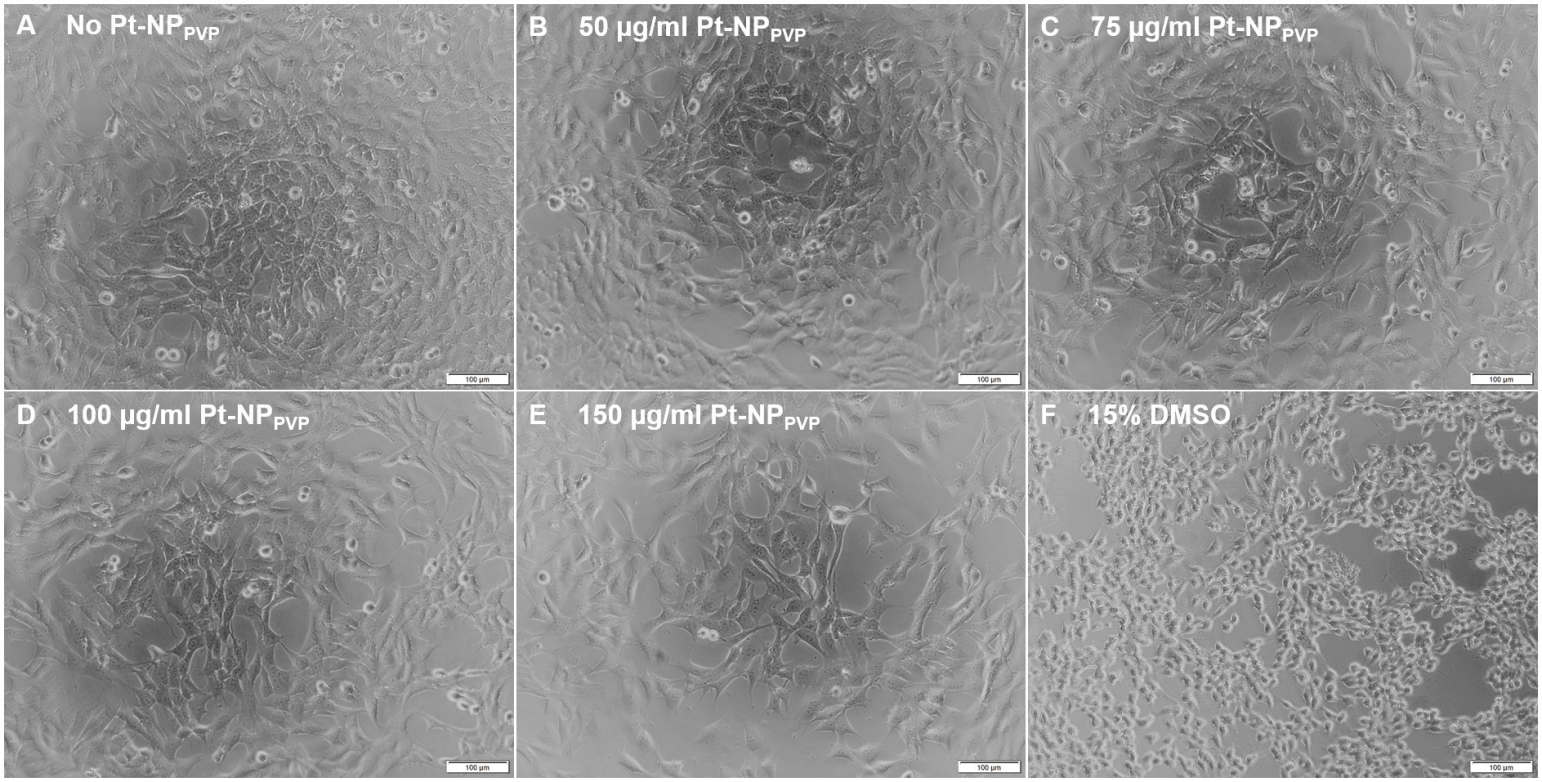
Microscopic view of the morphology of the HEI-OC1 cells following exposure to Pt-NP_PVP_. HEI-OC1 cells were cultivated either without Pt-NP_PVP_ administration as reference (A) or in culture medium containing 50 μg/ml (B), 75 μg/ml (C), 100 μg/ml (D) and 150 μg/ml (E) of the Pt-NP_PVP_. The cell culture assay exposed to 15% DMSO for 1.5 h was used as negative control (F). The transmission light images demonstrated highly uniform cell adhesion without any morphological damage throughout the cell cultures assays with and without varying Pt-NP_PVP_ concentrations. Size of the bars: 100 μm.

Following supplementation of the HEI-OC1 cell culture assays with varying Pt-NP_PVP_ concentrations between 50 μg/ml and 150 μg/ml the morphology of the cells was microscopically characterized. Representative microscopic views of the cell morphology of HEI-OC1 cells without Pt-NP_PVP_ administration (Fig 1A) as well as those exposed to Pt-NP_PVP_ up to 150 μg/ml revealed normal cell adhesion and growth without any signs of morphological irregularities (Fig 1B–1E), in comparison to the cell culture assay applied with DMSO serving as negative control.

**Fig 2:**
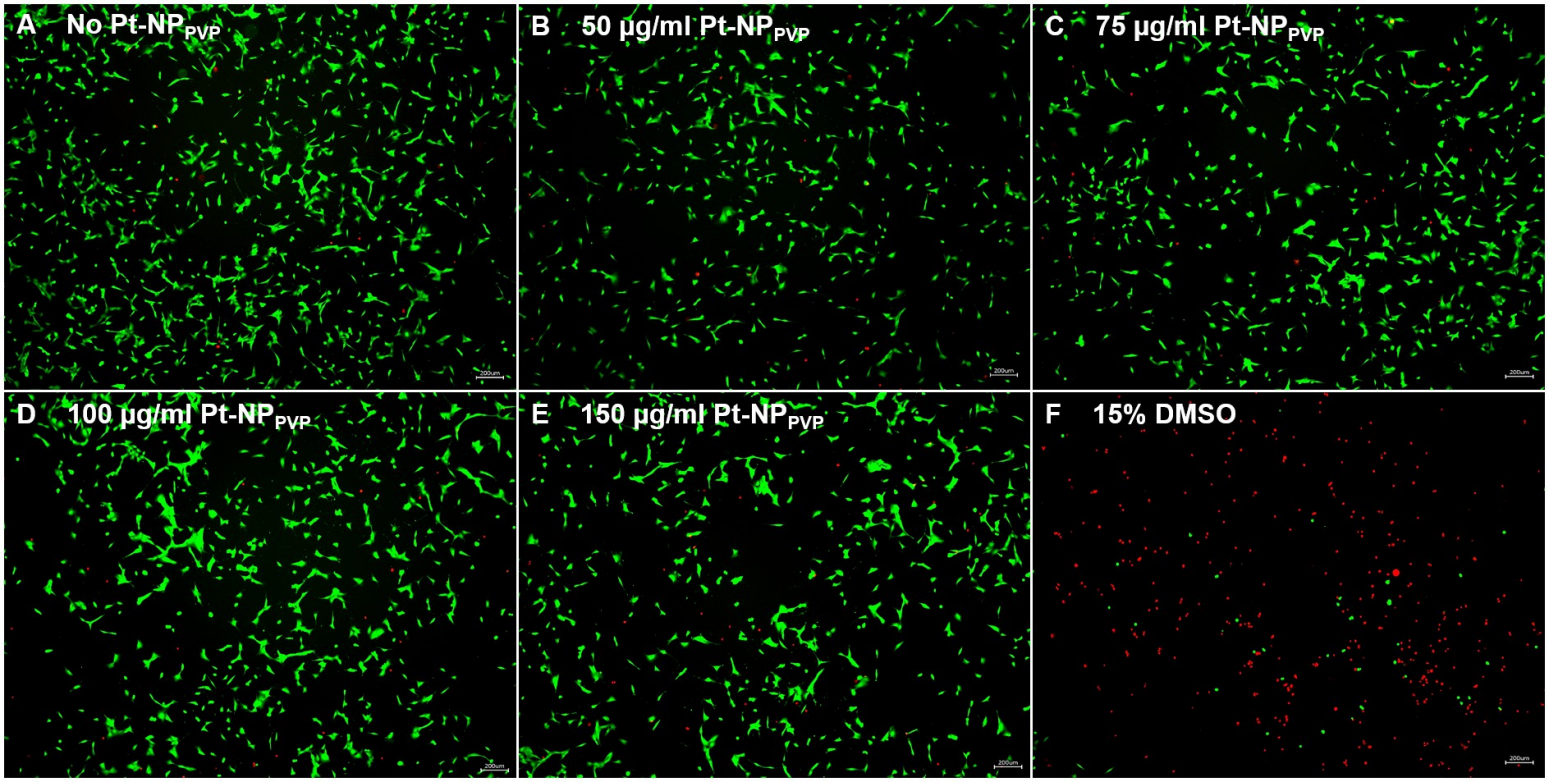
Fluorescent live cell staining of the HEI-OC1 cells with Calcein AM and EthDIII to distinguish between vital and dead cells following exposure to Pt-NP_PVP_. To distinguish between live and dead cells in the same cell population following Pt-NP_PVP_ administration Calcein AM (green) and EthD (red) were used for live cell staining. After permeating the cell membrane Calcein AM is cleaved to calcein by the esterases in the cytoplasm which turns to the green fluorescent dye by complexing Ca^2+^-ions. In contrast, EthD only enters dead cells, when their plasma membrane is disrupted to intercalate between two adenine–thymine base pairs resulting in strong red fluorescence. HEI-OC1 cells cultivated under standard conditions served as reference (A). Cell culture assays exposed to 50 μg/ml (B), 75 μg/ml (C), 100 μg/ml (D) and 150 μg/ml (E) Pt-NP_PVP_ revealed no nanoparticle induced cell death. As like in the cell culture assay without Pt-NP_PVP_ supplementation (A) the majority of the HEI-OC1 cells demonstrated Calcein AM induced green fluorescence indicating membrane integrity and normal growth (B-E). The presence of few red stained cells were related to processes inducing membrane disruption and, thus, allowing EthD to intercalate the DNA-strands as especially demonstrated by the cell culture assay supplied with DMSO serving as negative control (F). Size of the bars: 200 μm.

These results were confirmed by live cell staining with Calcein AM and EthD: Whereas Calcein AM represents viable cells following cleavage to calcein within the cytosol by esterases, EthD is only able to bind to the DNA of dying cells resulting in red fluorescence. Similar to the HEI-OC1 cell culture assay without Pt-NP_PVP_ supplementation (Fig 2A) those exposed to varying Pt-NP_PVP_ concentrations did not show any signs of cytotoxicity (Fig 2B–2E). As presented in Fig 2 the majority of the HEI-OC1 cells demonstrated Calcein AM induced green fluorescence indicating membrane integrity and normal growth. Only few red stained cells were found as the result of the formation of the EthD-DNA complexes due to the lack of the membrane integrity. In contrast to the live cell staining of the samples supplied with Pt-NP_PVP_ those exposed to DMSO showed opposite results: Nearly all cells underwent disintegration of the plasma membrane following DMSO incubation as presented by strong red fluorescence (Fig 2F).

**Fig 3:**
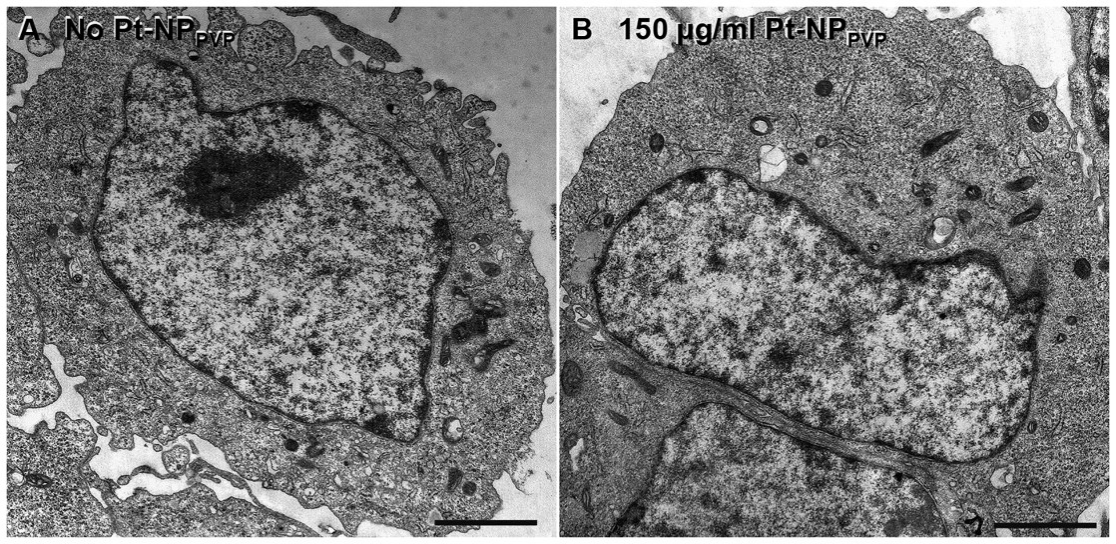
Ultrastructure of the HEI-OC1 cells following exposure to Pt-NP_PVP_. HEI-OC1 cells were cultivated either without Pt-NP_PVP_ incubation as reference (A) or in culture medium containing 150 μg/ml of the Pt-NP_PVP_ (B). No cytotoxic effect of Pt-NP_PVP_ could be found. The HEI-OC1 cells contained a few mitochondria und single endoplasmic reticulum profiles. Their endocytic activity was low. No Pt-NP_PVP_ were recognized on the cell surface or inside the cytosol. Size of the bars: 2 μm.

In accordance with the intact cellular morphology no ultrastructural changes were found in the HEI-OC1 cells in dependence of the Pt-NP_PVP_ concentration. Pt-NP_PVP_ could not be recognized on the surface or inside the cytosol of the cells. Interestingly, only a few mitochondria, short profiles of the endoplasmic reticulum as well rare endosomes were present (Fig 3).

**Fig 4:**
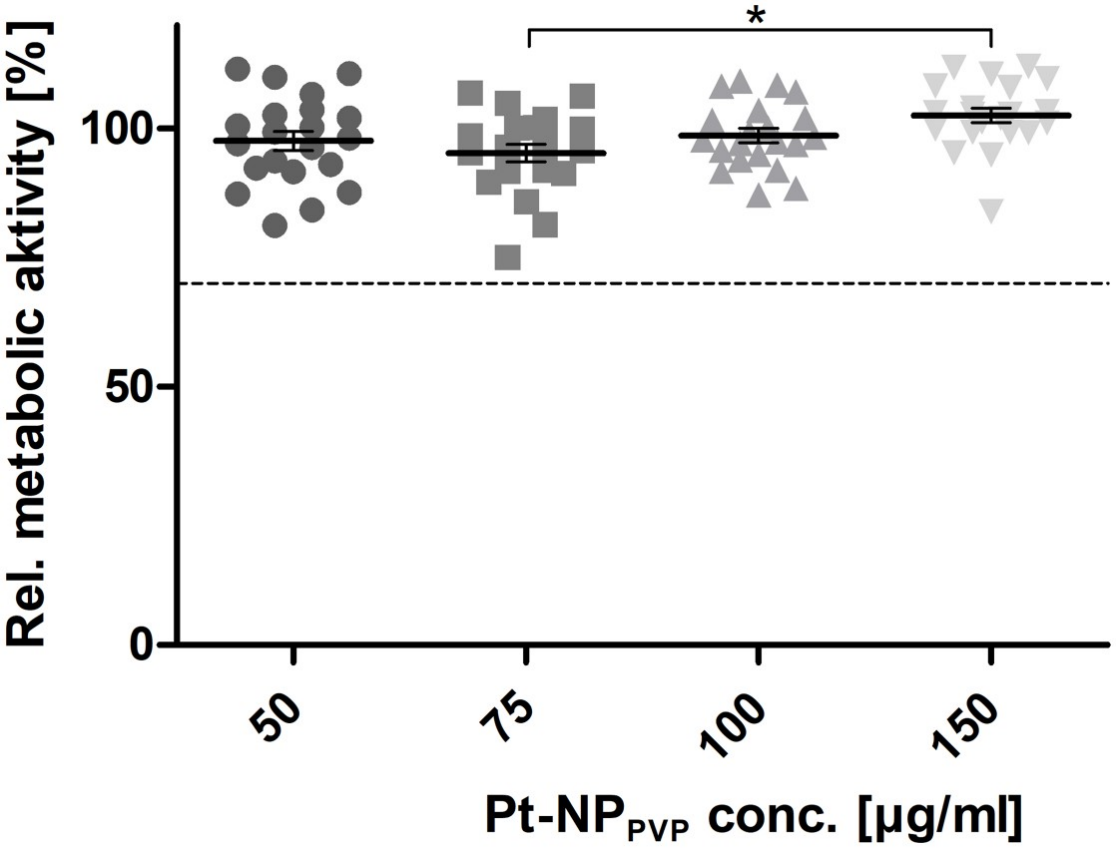
Determination of the metabolic activity of the HEI-OC1 cells following exposure to Pt-NP_PVP_. Metabolic activity of the HEI-OC1 cells grown in culture medium containing 50 μg/ml -150 μg/ml Pt-NP_PVP_ was determined by indirect dehydrogenases driven reduction of resazurin to the highly fluorescent resorufin. Fluorescence units (FU) were measured in 48 h cultivation assays (N = 6 independent experiments, n = 18). The resulting fluorescence intensities were also related to those obtained from the reference and calculated as percentage [%]. Each data point is presented as mean and SEM. One-way ANOVA with Tukey’s multiple comparison test was performed for statistical assessment (*p ≤ 0.05). The dashed line represents the cytotoxicity limit (70%).

Also, the characterisation of the metabolic activity revealed no cell death induction in the HEI-OC1 cells even at Pt-NP_PVP_ concentrations up to 150 μg/ml (Fig 4). The data demonstrated slight, but not significant, decrease of the metabolic activity following exposure to 50 μg/ml (93.68% ± 2.93), 75 μg/ml Pt-NP_PVP_ (89.99% ± 3.35) and 100 μg/ml (95.13% ± 2.91), followed by another increase with 150 μg/ml (102.72% ± 2.73) of the Pt-NP_PVP_.

### Survival rate and neurite growth of the SG neurons were not affected by Pt-NP_PVP_ up to 100 μg/ml

**Fig 5:**
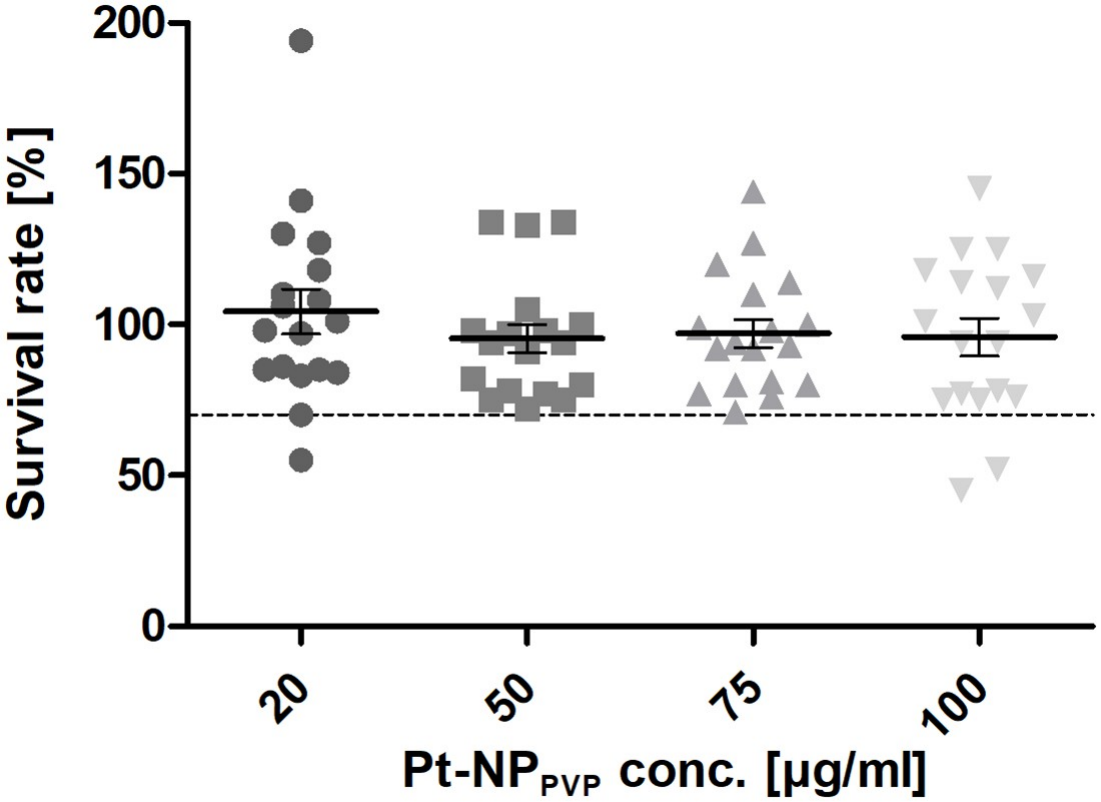
Determination of dose-dependent effects of Pt-NP_PVP_ on the survival rate and neurite outgrowth of the SG neurons cultivated for 48 h. Determination of the impact of varying Pt-NP_PVP_ concentrations on the survival rate (Fig 5) and neuritogenesis of the SG neurons characterized by anti-neurofilament 200 staining (Fig 6) cultivated for 48 h in cell culture medium containing 20 μg/ml—100 μg/ml Pt-NP_PVP_. Each data point is presented as mean and SEM of the (Fig 5) percentage of stained SG neuron soma in cell culture assays supplied with Pt-NP_PVP_ (N = 6, n = 18 of each Pt-NP_PVP_ concentration) in relation to the reference without Pt-NP_PVP_ administration and (Fig 6) length of nerve fibres (N = 6, n = 90 neurons exposed to varying Pt-NP_PVP_ concentration, respectively). One-way ANOVA with Tukey’s multiple comparison test was performed for statistical assessment. The dashed line in (Fig 5) represents the cytotoxicity limit (70%).

**Fig 6:**
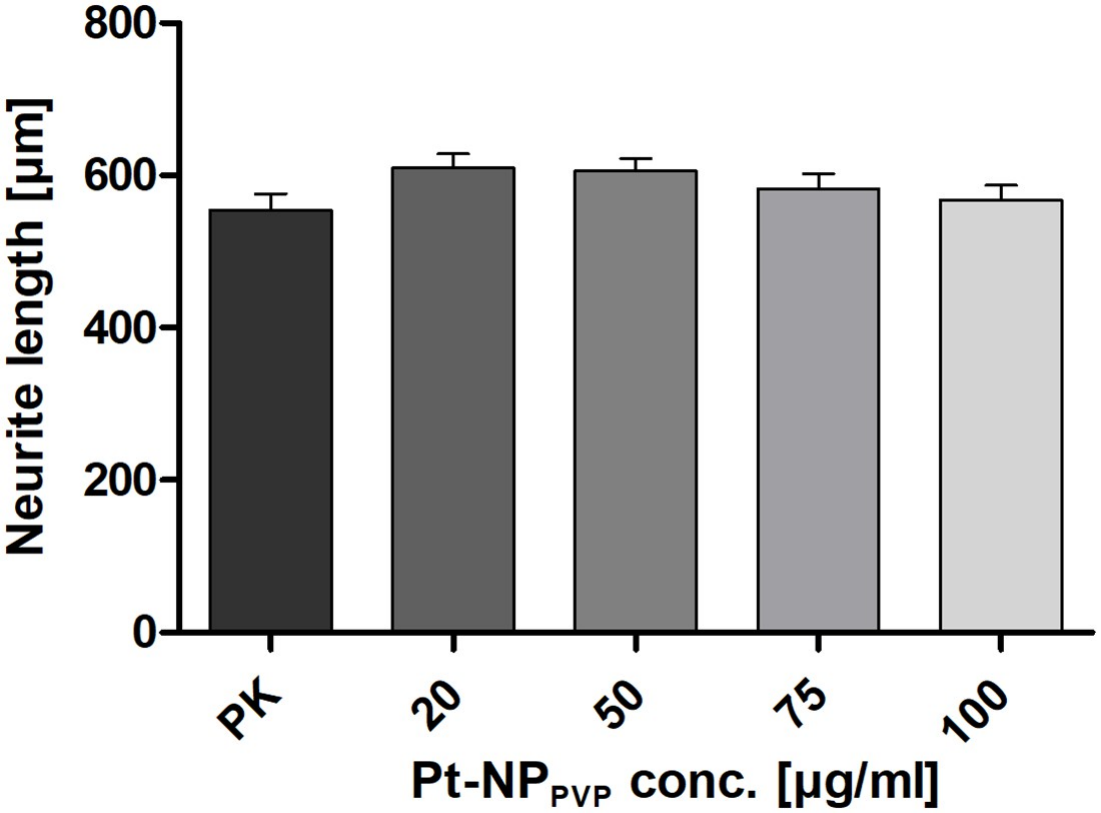
Determination of dose-dependent effects of Pt-NP_PVP_ on the survival rate and neurite outgrowth of the SG neurons cultivated for 48 h. Determination of the impact of varying Pt-NP_PVP_ concentrations on the survival rate (Fig 5) and neuritogenesis of the SG neurons characterized by anti-neurofilament 200 staining (Fig 6) cultivated for 48 h in cell culture medium containing 20 μg/ml—100 μg/ml Pt-NP_PVP_. Each data point is presented as mean and SEM of the (Fig 5) percentage of stained SG neuron soma in cell culture assays supplied with Pt-NP_PVP_ (N = 6, n = 18 of each Pt-NP_PVP_ concentration) in relation to the reference without Pt-NP_PVP_ administration and (Fig 6) length of nerve fibres (N = 6, n = 90 neurons exposed to varying Pt-NP_PVP_ concentration, respectively). One-way ANOVA with Tukey’s multiple comparison test was performed for statistical assessment. The dashed line in (Fig 5) represents the cytotoxicity limit (70%).

For the first time primary SG cells, especially its neurons, have been established as cell culture model for investigating potential Pt induced cytotoxicity. Supposing a higher sensitivity of the neurons to Pt-NP_PVP_ its concentration series was set from 20 μg/ml up to 100 μg/ml. As presented in Fig 5 no significant Pt-NP_PVP_ induced reduction of the survival rate of the SG neurons, specifically characterized with anti-neurofilament 200-antibody, in comparison to the reference without nanoparticle administration could be detected. However, our data revealed a clear tendency of a decrease of the SG neuron survival. Whereas 20 μg/ml (103.65% ± 6.3) of Pt-NP_PVP_ did not show any effects on the neuronal viability, the higher Pt-NP_PVP_ concentrations from 50 μg/ml (91.19% ± 5.9) and 75 μg/ml (91.34% ± 5.02) up to 100 μg/ml (85.34% ± 5.32) seemed to impair their cell metabolism and adhesion (Fig 5). In contrast, the determination of the neurite outgrowth in the context of dose-dependent Pt-NP_PVP_ administration did not reflect the results of the examination of the survival rates found in the cultivation assays exposed to the same Pt-NP_PVP_ concentration. As shown in Fig 6 any Pt-NP_PVP_ concentration had an impact on the outgrowth of the neurites: The untreated SG neurons yielded an average neurite length of 534.76 μm (± 17.43 μm), followed by neurite length of 610.16 μm (± 18.35 μm), 605.37 μm (± 17.19 μm), 556.84 μm (± 16.72 μm) and 539.67 (± 16.84 μm) measured in SG cell cultivation assays containing 20, 50, 75 and 100 μg/ml, respectively.

**Fig 7:**
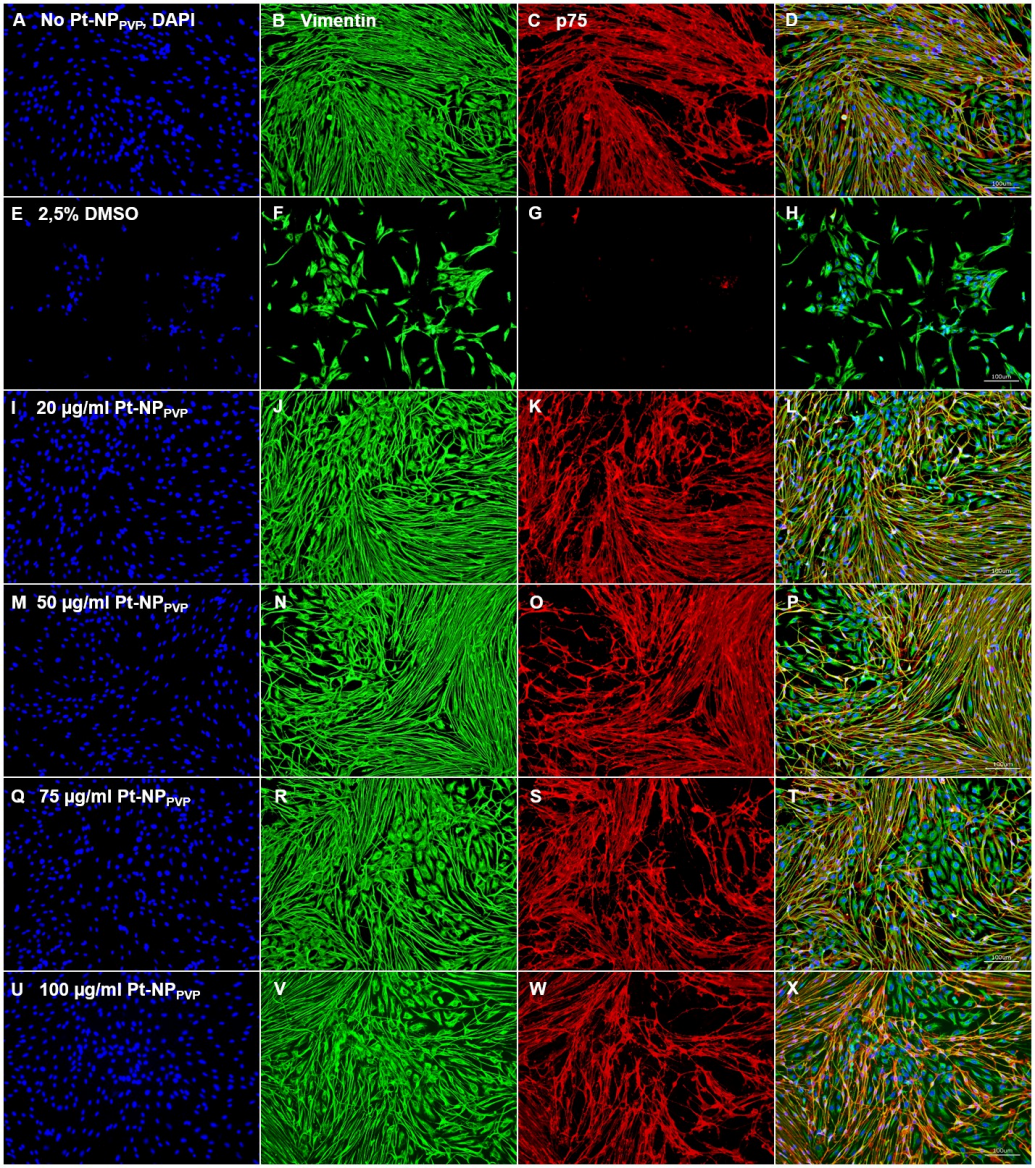
Representative fluorescence images of immunostained SG cells following Pt-NP_PVP_ application for 48 h. Representative fluorescent microscopic views of non-neuronal cells, especially fibroblasts and glial cells, labelled with anti-vimentin (Vim, green) and anti-p75 neurotrophic growth factor receptor (p75-NGFR, red) antibody, respectively. Nuclei were stained with DAPI. Positive staining against vimentin and p75-NGFR antigens were found in cell culture assays without Pt-NP_PVP_ incubation (A-D) and supplied with 20 μg/ml (I-L), 50 μg/ml (M-P), 75 μg/ml (Q-T) and 100 μg/ml Pt-NP_PVP_ (U-X). Staining of the fibroblasts and glial cells showed vital cells and no morphological irregularities even at the highest Pt-NP_PVP_ concentration. Cells treated with 2.5% DMSO (E-H) were used as negative control. Size of the bars 100 μm.

**Fig 8:**
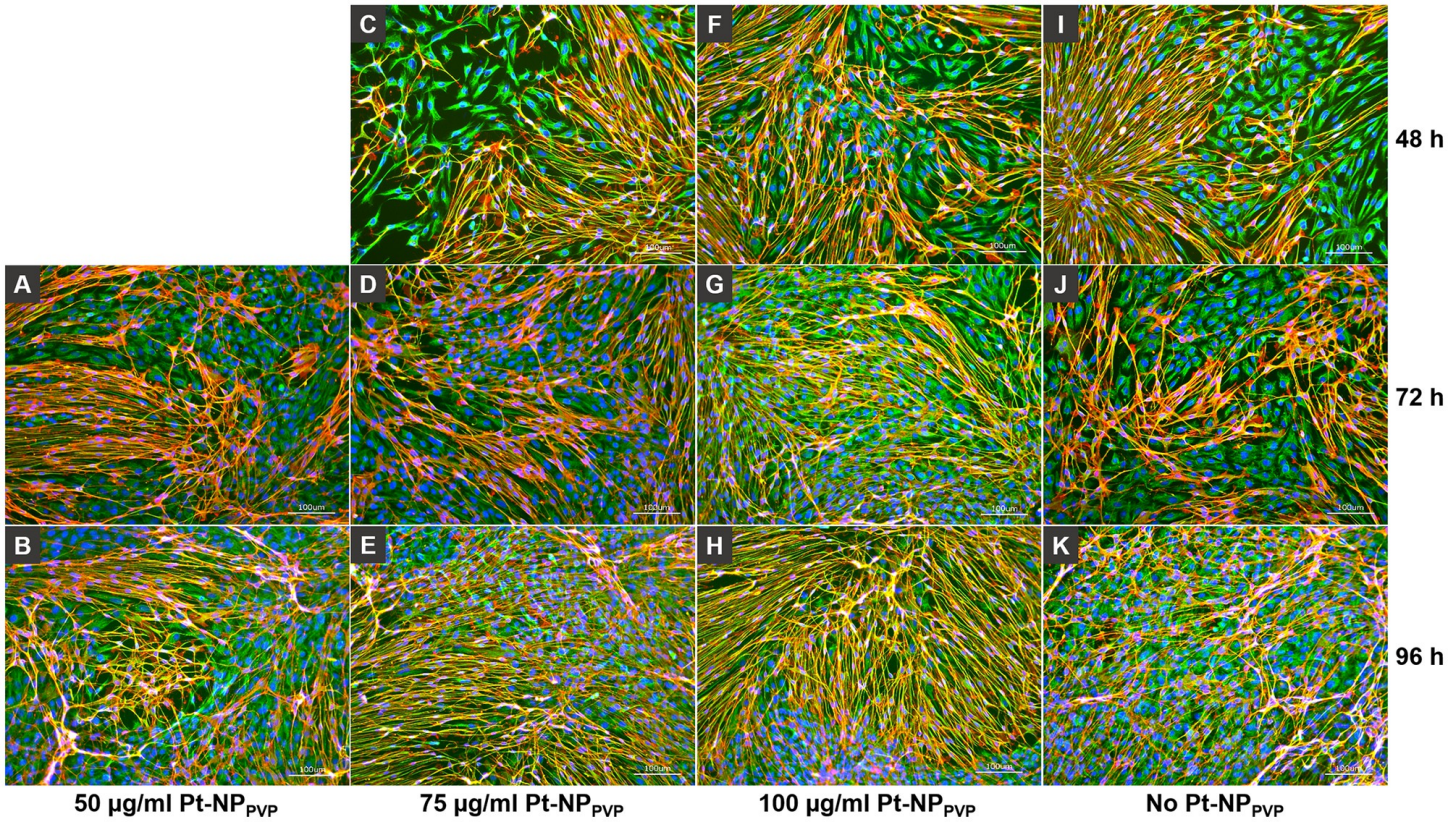
Representative fluorescence images of immunostained SG cells following Pt-NP_PVP_ application for 48 h, 72 h and 96 h. Representative fluorescent microscopic views of fibroblasts and glial cells, labelled with anti-vimentin (Vim, green) and anti-p75 neurotrophic growth factor receptor (p75-NGFR, red) antibody, respectively. Nuclei were stained with DAPI. Positive staining against vimentin and p75-NGFR antigens were found in cell culture assays without Pt-NP_PVP_ incubation (I-K) and supplied with 50 μg/ml (A-B) for 72 h and 96 h cultivation, 75 μg/ml (C-E) and 100 μg/ml Pt-NP_PVP_ (F-H) for 48 h, 72 h and 96 h cultivation, respectively. Staining of the fibroblasts and glial cells showed widespread cell adhesion and growth not only at the highest Pt-NP_PVP_ concentration, but also independently of the cultivation period. Size of the bars 100 μm.

The composition of the SG cell population was characterized by specific antigen staining after Pt-NP_PVP_ incubation. We found positive staining against vimentin and p75-NGFR antigens in cell culture assays with and without Pt-NP_PVP_ administration. As shown in Fig 7 neither the fibroblasts nor the glial cells demonstrated any signs of cell death induction, instead widespread cell adhesion und normal morphological appearance even at the highest Pt-NP_PVP_ concentration (Fig 7I–7X). In contrast, the SG cells exposed to 2.5% DMSO demonstrated strong decrease in both fibroblast and glial cell growth, especially only few glial cells could be detected (Fig 7E–7H). By these findings the question arose if longer culture periods with the same Pt-NP_PVP_ concentrations may induce cytotoxic mechanisms rather than 48 h cultivation alone. Despite the longer cultivation period up to 72 h and the exposition to the highest Pt-NP_PVP_ concentration no decrease of the cell density or changes in adhesion behaviour and cell morphology could be detected (Fig 8). Indeed, double staining of the fibroblasts and glial cells revealed similar to the reference without Pt-NP_PVP_ application normal cell growth and morphological appearance throughout the increasing Pt-NP_PVP_ and cultivation period.

## Discussion

In contrast to our previous study of the impact of Pt-NP_PVP_ on the metabolism of NIH 3T3 and human neuroblastoma cells (SH-SY5Y) [50] the HEI-OC1 cell culture model did not demonstrate any significant influence of the Pt-NP_PVP_ on the cell viability even at higher concentrations up to 150 μg/ml. Microscopical evaluation and relative quantification of the metabolic activity confirmed no changing of the cell morphology, cell adhesion and growth following Pt-NP_PVP_ administration. Also, the differential live cell staining with Calcein AM indicated membrane integrity for the majority of the HEI-OC1 cells, whereas the presence of few red stained cells were related to processes inducing membrane disruption and, thus, allowing EthD to intercalate the DNA-strands. By these findings, it can be concluded that Pt-NP_PVP_ used in this study do not induce cell death related signal pathways in comparison to the cell culture assays exposed to DMSO which served as negative control. Similar results were obtained from the cell culture model of the spiral ganglion cells: Neither dose- nor time-dependent decrease of both the SG neuron survival and neurite extension could be observed following Pt-NP_PVP_ supplementation. As described by Hadler et al. [55] SG neuron survival is limited in association with a decline of Schwann cells and other glial cells due to the failing trophic support. These cells are involved in promoting neuronal survival, growth and regeneration by providing myelination and trophic support and also by expressing cell surface and extracellular matrix proteins [56, 57]. In this study positive staining of the intermediary filament vimentin (Vim) and the neurotrophic growth factor receptor (p75-NGFR) showed widespread adhesion und normal morphology of both the fibroblasts and glial cells even at the highest Pt-NP_PVP_ concentration. Interestingly, the examination of the effects of Pt-NP_PVP_ throughout a longer cultivation period up to 96 h did not visualize any cell death induction as demonstrated by the normal growth of the fibroblasts and the glial cells. These observations indicate that Pt-NP_PVP_ do not disturb membrane plasticity and induce cell death mechanisms in the spiral ganglion cell cultures. Instead, the glial cells seem to be able to maintain neuronal vitality and to facilitate adhesion and neurite extension of the SG neurons in the first 48 h culture period. At all, our study did not confirm the hypothesis that Pt-NP_PVP_ supplementation to the inner ear cell culture model initiate decrease in metabolic activity, cell growth and survival as well as neurite growth.

So far, our results seemed to be in line with *in vitro* studies exposing numerous animal and human cell lines to Pt-NP with less than 10 nm in particle size and within a certain concentration range: No or moderate cell death induction have been described up to 80 μg/ml [31–36, 41, 49], only higher Pt-NP concentrations exposed to both healthy and tumor cells induced stress and DNA damage [31–33, 49, 50]. It is well known, that Pt-NP may also display enzymological properties for scavenging H_2_O_2_ and superoxide anion radicals [37–41, 58, 59], which could be demonstrated in tests with Pt-NP sizes from 1 to 5 nm in several cell lines. Hereby, 1 nm Pt-NP showed the highest ability for scavenging of reactive oxygen species and, thus, no cytotoxicity could be confirmed at Pt-NP concentrations as high as 50 μg/ml [30].

The question is, by which cellular, physico-chemical and nanoparticle manufacturing conditions the biological effects of the Pt-NP can be deduced. As previously reviewed [60, 61], their cellular uptake and biological effects is determined not only by cell and tissue type presenting specific compositions of surface proteins and surface charge, but also by particle size, agglomeration, protein absorption or capping of the Pt-NP with polymeric compounds [33, 50, 62, 63]. These parameters may influence the interactions of the Pt-NP with the membrane and, thus, the translocation mechanisms and cytotoxicity. For this study Pt-NP was provided as colloidal solution stabilized with polyvinylpyrrolidone (PVP) to impair agglomeration of the particles and ensure effective dispersion [63, 64]. As shown by transmission electron microscope accumulation of large Pt-NP_PVP_ aggregates on the surface of the pelletized HEI-OC1 cells could not be detected indicating uniform distribution in the cell culture medium.

As discussed above PVP coated Pt-NP demonstrated cell type dependent biological effects: Whereas no changes in cell metabolism of inner ear related cells could be found, our previous study with NIH 3T3 and SH-SY5Y cells supplemented with the same Pt-NP_PVP_ presented contradictory results [50]. Similarly, Demir et al. confirmed any adverse biological effects in fish cells following exposure to PVP coated Pt-NP with particle size around 4–9 nm, whereas those without any coating induced cytotoxicity and genotoxicity in fish cell assays at concentrations above 75 μg/ml [49]. However, another study reported that PVP protected Pt-NP with 5.8 nm in particle size exposed to human epidermal keratinocytes in concentrations up to 25 μg/ml triggered the decrease of cell metabolism and DNA stability [65]. Those controversial behavior of the Pt-NP may be the consequences of the interactions of the PVP capped Pt-NP either with the membrane surface charge or with specific membrane proteins and receptors.

To unfold either their cell protection or damaging effects the Pt-NP needs to be taken up by the target cells. Depending on the Pt-NP size, they were found to penetrate the cells either by diffusion or through endocytosis by enclosing of the nanoparticles into the lysosomes [31–33, 60–61, 65, 66]. As a result of the endocytotic cell entry, accumulation of the nanoparticles inside the lysosomes lead to the degradation of particular Pt due to low pH and degradative enzymes in the lysosomes [65, 67, 68]. It is suggested that apoptosis/necrosis is initiated through the combined effects of Pt-NPs and Pt^2+^ released from the nanoparticles causing ROS production and DNA damage [31]. As previously reported in Wissel et al. dose-dependent internalization of Pt-NP_PVP_ into the multivesicular bodies of the NIH 3T3 cells could be proven, but no diffusion into the cytosol or nucleus was visible. Interestingly, the SH-SY5Y cells did not enclose any Pt-NP_PVP_, even though decrease of cell growth and metabolic activity have been proven [50]. As also described in this study HEI-OC1 cells exposed to 150 μg/ml Pt-NP_PVP_ disclosed ultrastructurally no Pt-NP_PVP_ inside the cytosol. In the consequence Pt-NP is not able to trigger cell damage–if they are capped with polymeric compounds.

Despite the fact that Pt-NP may not be harmful for the spiral ganglion cells, it has to be considered that electrical stimulation over years may either accumulate Pt particles around the cell membranes causing disturbances in intercellular communications or initiate internalization into other inner ear cell types inducing oxidative stress. In both cases particular Pt may indirectly compromise auditory nerve plasticity and, thus, induce cell death signaling. On the other side, the results of the animal studies conducted by the group of Shepherd [20–22] indicated that within the cochlea systemic Pt toxicity derived from corroding electrode surfaces, even under extremely high charge density stimulation, appear not to be of clinical significance. This conclusion was underlined by the findings that the majority of particulate Pt not only had undergone phagocytosis within the scala tympani, but also remained bound within macrophages over years [25, 69]. However, other electrochemical reaction products may alter the electrochemical environment at the electrode–tissue interface, initiating foreign body response and immune cascades [20–22, 69]. Moreover, it is more likely that ionic corrosion products as like as Pt ions may be transferred within the cochlear tissues inducing cell death pathways.

## Conclusion

In this study cochlear derived cells–either as the mouse organ of Corti cell line (HEI-OC1) or as rat primary spiral ganglion cells–were used for the first time as *in vitro* cell culture model for the evaluation of the cytotoxic potential of commercially manufactured Pt-NP_PVP_. In contrast to the results achieved from the cell culture model of both NIH 3T3 and SH-SY5Y cells no decrease of metabolic activities, cell growth and survival as well as neurite outgrowth was found. Furthermore, no changings in cell adherence and morphology have been detected at even high Pt-NP_PVP_ concentrations up to 150 μg/ml. Immunocytochemical evaluation revealed normal cell spreading of the SG cells in both dose- and time-dependent Pt-NP_PVP_ exposure up to 96 h. Since in HEI-OC1 no lysosomal Pt-NP_PVP_ enrichment could be demonstrated ultrastructurally, it can be concluded that the Pt-NP_PVP_ used in this study do not trigger their cellular uptake and, thus, cannot initiate apoptotic pathways in the HEI-OC1 cell line as well in the SG cells.

Transforming these findings to the implanted cochlea electrode particular corrosion products may not be able to unfold potential cytotoxicity in the organ of Corti or the auditory nerve, unless they are not passing the cell membrane. However, it has to be considered that electrical stimulation over years may either accumulate Pt particles around the cell membranes causing disorders in intercellular communications, unless they are engulfed in phagosomes of macrophages and giant cells within the electrode tract. Additionally, depending on their size Pt particles may trigger internalization into special inner ear cell types inducing cell damage. Hence, in both cases auditory nerve plasticity may be indirectly interfered.

## Supporting information

S1 Fig Ultrastructure of the HEI-OC1 cells following exposure to 50 μg/ml, 75 μg/ml and 100 μg/ml Pt-NP_PVP_. HEI-OC1 cells cultivated in culture medium containing 50 μg/ml (A), 75 μg/ml (B) and 100 μg/ml Pt-NP_PVP_ (C) demonstrate no cytotoxic sign. Nevertheless a few mitochondria delivered the energy for the synthetic activity of the cells in the endoplasmic reticulum as well inside the cytosol. Size of the bars: 2 μm.
(TIF)

S1 Data Minimal underlying dataset of the study. (PDF)

## Abbreviations

Calcein AM: calcein acetoxymethyl
CI: Cochlear implant
EthD: ethidium homodimer III
HEI-OC1: organ of Corti cell line from the Hearing-Ear-Institute-Organ
Pt-NP: platinum nanoparticles
Pt-NP_PVP_: PVP coated platinum nanoparticles
PVP: polyvinylpyrrolidone
SEM: Standard error of mean
SG: spiral ganglion
