# Supplementary figures and images for: In vitro impact of platinum nanoparticles on inner ear related cell culture models
Source: PLoS One. 2023 Apr 24;18(4):e0284794. doi: 10.1371/journal.pone.0284794 (PMC10124869; doi:10.1371/journal.pone.0284794)

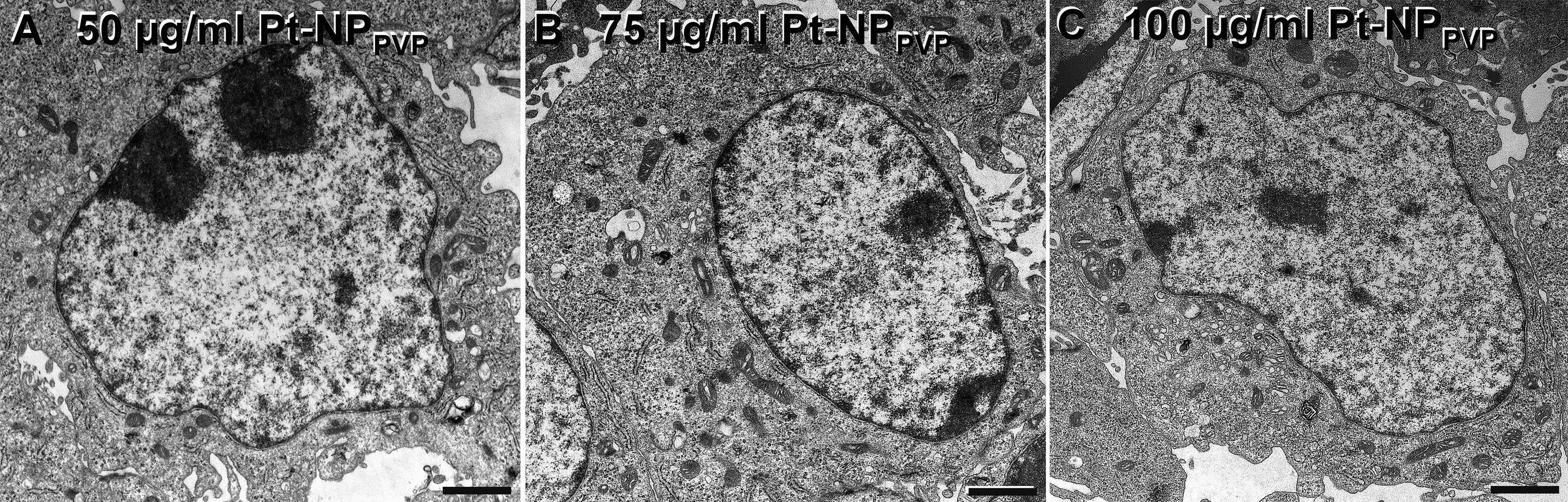

Supplement: S1 Fig — HEI-OC1 cells cultivated in culture medium containing 50 μg/ml (A), 75 μg/ml (B) and 100 μg/ml Pt-NPPVP (C) demonstrate no cytotoxic sign. Nevertheless a few mitochondria delivered the energy for the synthetic activity of the cells in the endoplasmic reticulum as well inside the cytosol. Size of the bars: 2 μm. (TIF) [file pone.0284794.s001.tif]
