# Supplementary material for: In vitro impact of platinum nanoparticles on inner ear related cell culture models
Source: PLoS One. 2023 Apr 24;18(4):e0284794. doi: 10.1371/journal.pone.0284794 (PMC10124869; doi:10.1371/journal.pone.0284794)
Supplement: S1 Data — (PDF) [file pone.0284794.s002.pdf]

MINIMAL DATA SET

Cell viability assay of HEI-OC1 cells following Pt-NP_{PVP} incubation

50 µg/ml	75 µg/ml	100 µg/ml	150 µg/ml
104,46 % ± 3,15	92,46 % ± 2,03	90,5 % ± 2,69	98,84 % ± 7,84
109,38 % ± 1,46	102,23 %± 1,44	96,77 % ± 3,5	101,41 % ± 3,78
96,01 % ± 4,75	98,94 % ± 3,73	105,08 % ± 3,64	104,47 % ± 2,05
98,25 % ± 1,11	97,18 % ± 0,97	99,05 % ± 1,33	102,02 % ± 1,07
87,87 & ± 2,13	86,41 % ± 3,02	99,94 % ± 4,38	100,45 % ± 2,58
98,35 % ± 2,82	99,65 % ± 4,42	103,11 % ± 2,1	102,73 % ± 3,44
89,1 % ± 3,94	89,9 % ± 7,56	96,1 % ± 2,18	107,77 % ± 4,21

Data are presented as the mean of the triplicates normalized against the untreated cells.

Survival rate of SG neurons following Pt-NP_{PVP} incubation

20 µg/ml	50 µg/ml	75 µg/ml	100 µg/ml
118,95 % ± 11,17	89,54 % ± 7,71	113,07 % ± 10,7	98,04 % ± 14,85
103,67 % ± 13,88	92,35 % ± 5,36	107,95 % ± 18,96	96,33 % ± 12
109,82 % ± 12,85	101,23 % ± 17,4	100,61 % ± 5,35	124,54 % ± 10,48
95,31 % ± 4,56	91,56 % ± 10,12	85,28 % ± 6,51	96,93 % ± 10,98
70,03 % ± 8,74	82,57 % ± 4,2	85,93 % ± 7,66	101,83 % ± 13,51
128,79 % ± 33,48	115,15 % ± 18,37	89,77 % ± 11,97	57,58 % ± 8,93

Data are presented as the mean of the triplicates normalized against the untreated cells.

Neurite outgrowth of SG neurons following Pt-NP_{PVP} incubation

PK	20 µg/ml	50 µg/ml	75 µg/ml	100 µg/ml
472,24 µm ± 19,17	563,47 µm ± 33,91	548,83 µm ± 40,9	433,92 µm ± 25,13	448,68 µm ± 29,82
442,15 µm ± 27,24	576,1 µm ± 35,8	505,34 µm ± 29,95	525,51 µm ± 26,79	548 µm ± 48,21
500,22 µm ± 32,89	680,73 µm ± 35,29	647,75 µm ± 30,35	681 µm ± 45,62	650,7 µm ± 20,25
507,95 µm ± 44,46	551,33 µm ± 40,94	555,58 µm ± 28,76	524,41 µm ± 28,51	454,64 µm ± 23,18
732,49 µm ± 77,61	627,36 µm ± 42,73	650,45 µm ± 37,66	603,98 µm ± 50,9	628,42 µm ± 45,72
830,8 µm ± 40,66	782,2 µm ± 75,37	852,78 µm ± 34,08	906,26 µm ± 37,48	848,25 µm ± 46,81
394,65 µm ± 22,65	489,92 µm ± 25,33	476,89 µm ± 39,09	400,96 µm ± 21,69	394,09 µm ± 34,46

Data are presented as the mean of the triplicates.
